# Comparative genomic analysis of *Ralstonia solanacearum* reveals candidate avirulence effectors in HA4-1 triggering wild potato immunity

**DOI:** 10.3389/fpls.2023.1075042

**Published:** 2023-02-23

**Authors:** Mengshu Huang, Xiaodan Tan, Botao Song, Yuqi Wang, Dong Cheng, Bingsen Wang, Huilan Chen

**Affiliations:** ^1^ National Key Laboratory for Germplasm Innovation & Utilization of Horticultural Crops, Huazhong Agricultural University, Wuhan, Hubei, China; ^2^ Key Laboratory of Potato Biology and Biotechnology (HZAU), Ministry of Agriculture and Rural Affairs, Wuhan, Hubei, China; ^3^ College of Horticulture and Forestry Science, Huazhong Agricultural University, Wuhan, Hubei, China; ^4^ Guangdong University Key Laboratory for Sustainable Control of Fruit and Vegetable Diseases and Pests & Key Laboratory of Green Prevention and Control on Fruits and Vegetables in South China, Ministry of Agriculture and Rural Affairs, Zhongkai University of Agriculture and Engineering, Guangzhou, China

**Keywords:** *Ralstonia solanacearum*-potato interaction, bacterial resistance, avirulence effector, comparative genomics, type III effectors

## Abstract

*Ralstonia solanacearum* is the causal agent of potato bacterial wilt, a major potato bacterial disease. Among the pathogenicity determinants, the Type III Secretion System Effectors (T3Es) play a vital role in the interaction. Investigating the avirulent T3Es recognized by host resistance proteins is an effective method to uncover the resistance mechanism of potato against *R. solanacearum*. Two closely related *R. solanacearum* strains HA4-1 and HZAU091 were found to be avirulent and highly virulent to the wild potato *Solanum albicans* 28-1, respectively. The complete genome of HZAU091 was sequenced in this study. HZAU091 and HA4-1 shared over 99.9% nucleotide identity with each other. Comparing genomics of closely related strains provides deeper insights into the interaction between hosts and pathogens, especially the mechanism of virulence. The comparison of type III effector repertoires between HA4-1 and HZAU091 uncovered seven distinct effectors. Two predicted effectors RipA5 and the novel effector RipBS in HA4-1 could significantly reduce the virulence of HZAU091 when they were transformed into HZAU091. Furthermore, the pathogenicity assays of mutated strains HA4-1 ΔRipS6, HA4-1 ΔRipO1, HA4-1 ΔRipBS, and HA4-1 ΔHyp6 uncovered that the absence of these T3Es enhanced the HA4-1 virulence to wild potato *S. albicans* 28-1. This result indicated that these T3Es may be recognized by *S. albicans* 28-1 as avirulence proteins to trigger the resistance. In summary, this study provides a foundation to unravel the *R. solanacearum*-potato interaction and facilitates the development of resistance potato against bacterial wilt.

## Introduction

Bacterial wilt disease, previously known as brown rot, is caused by the Gram-negative bacteria *Ralstonia solanacearum*. The *R. solanacearum* species complex (RSSC), one of the most harmful bacterial diseases that affect the production of many important crops worldwide, has a broad range of hosts varying from dicots to monocots. A report showed that RSSC could infect more than 250 plant species from 54 families in the world (Prior et al., 2016). According to the survey of the International Potato Center, bacterial wilt is one of the five biggest challenges affecting potato (*Solanum tuberosum* L.) yield in tropical and subtropical regions, which can account for up to 75% yield of losses (Rich, 1983; Wale et al., 2008; Patil et al., 2012). Some wild potato species and cultivated varieties are resistant or highly tolerant to bacterial wilt, such as *S. chacoense*, *S. microdontum*, *S. phureja*, *S. stenotonum*, and BR clones (the hybrid offspring containing the germplasms of *S. phuerja* and *S. demissum* are resistant to both bacterial wilt disease and late blight disease) and varieties (Machida-Hirano, 2015). The poor adaptability to various regions and the ineffectiveness against all strains of the pathogen hinder the wide application of these resistance resources (Prior et al., 1998; Wang et al., 2017). Taxonomists classified the RSSC into three species: *R. solanacearum* (phylotype II), *R. pseuosolanacearum* (phylotype I and III), and *R. syzygii* (phylotype IV) based on their phylotype and the geographical distribution (Genin and Denny, 2012; Safni et al., 2014).

Comparative genomics is a powerful tool for researchers to explore biological problems and explain biological phenomena. With the rapid increase in high-quality genomes, the application of comparative genomics is more and more extensive and in-depth (Setubal et al., 2018). So far, comparative genomics has been used in various species such as the Zoonotic pathogen *Ehrlichia chaffeensis*, revealing candidate effectors and putative host cell targets (Noroy and Meyer, 2017). The complete genome of seven highly virulent *Xanthomonas. translucens* strains have been obtained, and the T3Es were identified, giving an insight into the pathovar-specific population structure (Shah et al., 2021). The comparative genome analysis of *Clavibacter michiganensis*, the causal agent of bacterial canker in tomatoes, shows the distinct gene contents in the plasmids (Oh et al., 2022). The first *R. solanacearum* genome GMI1000 was sequenced in 2002 and has been widely utilized in *R. solanacearum* studies (Salanoubat et al., 2002). Up to now, over 300 *R. solanacearum* genomes have been sequenced and uploaded to NCBI (Genome List - Genome - NCBI), giving enriched information to uncover the similarities and differences among the strains. The comparative genome research of *R. solanacearum* has been divided into two ways: 1. A few genomes of the strains possess different pathogenicity to the host, such as PeaFJ1 and HA4-1 (Wan and Yang, 2022); 2. Meta-analysis of multiple genomes to discover the evolutionary relationships, phylogeny, and populations since individual strains vary in multiple conditions. For example, 100 RSSC genomes have been analyzed to highlight the evolution and diversity of *R. solanacearum* (Sharma et al., 2022). As many as 150 sequenced *R. solanacearum* strains have been used to analyze the T3Es repertoires, and the comparative genomics strategy has been proven to be useful with the identification of eight new T3Es and two new hypothetical T3Es (Sabbagh et al., 2019).

*R. solanacearum* possesses many kinds of virulent factors, such as the pili, exopolysaccharide (EPS), and genes that can express compounds to overcome host stress (Genin and Denny, 2012). Type III secretion system (T3SS) is one of the most important and generally acknowledged virulence factors utilized in plant-microbe interactions. T3SS is a syringe-like structure that delivers effectors into the cytoplasm or the plasma membrane of a plant cell (Deng et al., 2017). The proteins secreted from T3SS are called type III secretion system effectors (T3Es). *R. solanacearum* has more than 100 T3Es that can affect plant immunity by suppressing or triggering the plant defense response, targeting and modulating the plant metabolism by various molecular mechanisms (Landry et al., 2020). The methods of identifying T3Es are as followed: 1. Homology search using amino acid sequences of previously reported effectors; 2. Identification of the *hrpII* box (TTCGn16TTCG) or PIP (plant-induced promoter) box in the promoters (Cunnac et al., 2004); 3. Based on gene expression, such as the experiment using a Calmodulin-dependent adenylate cyclase (Cya) reporter system (Mukaihara et al., 2010); 4. Bioinformatics prediction approaches with gradually improved databases, such as “Ralsto T3E” (Peeters et al., 2013). Plant resistance against bacterial disease usually depends on the hosts successfully perceiving the avirulent effectors (Flor, 1971). More and more research on *R. solanacearum* avirulent effectors has been reported in *Solanum* species, such as RipJ, RipAX2, and RipAZ1 in tomato, eggplant, and black nightshade. The first avirulent gene to trigger bacterial resistance in tomato *Solanum pimpinellifolium* LA2093 was RipJ (Pandey et al., 2021). RipAZ1 confers avirulence in black nightshade *S. americanum* and triggers cell death on the leaves (Moon et al., 2021). RipAX2 is a highly prevalent effector avirulent in eggplant AG91-25, which possesses a major resistance locus *EBWR9* (Morel et al., 2018). However, the effectors of *R. solanacearum* that regulate and trigger potato resistance remain unclear.

Our previous study sequenced the genome of *R. solanacearum* strain HA4-1 and found that HA4-1 and another *R. solanacearum* strain, GMI1000, were avirulent and highly virulent to the wild potato species *Solanum albicans* 28-1 (ALB28-1), respectively (Tan et al., 2019). This result indicated that HA4-1 may contain avirulence effectors that could be recognized by resistant proteins in ALB28-1. In this study, we found that a novel *R. solanacearum* strain HZAU091 isolated from potato also displayed strong virulence to *Solanum albicans* 28-1. HZAU091 is more closely related to HA4-1 (PeaHuB4) than GMI1000 based on the genetic and pathogenic diversity analysis (Wang et al., 2017). To reveal the avirulence effectors triggering potato immunity, the complete genome of HZAU091 was sequenced to perform comparative genomic analysis and T3E repertoire analysis with HA4-1. Mutant strains and the virulent strain HZAU091 carrying effectors from HA4-1 were constructed to verify the candidate avirulence effectors by inoculation experiments.

## Materials and methods

### Plant materials and bacterial strains

The potato seedlings of *Solanum albicans* 28-1 (ALB28-1) were cultivated in Murashige-Skoog (MS) medium (supplemented with 4% sucrose and 0.75% agar) and grown for approximately 3 weeks *in vitro* at 20 ± 1°C with a 16 h/8 h (day/night) regime. The seedlings were then transferred into pots containing nutritional soil (ShangDao DB37/T1142-2008) and grown in a greenhouse at 22 ± 2°C. After 3-4 weeks, the seedlings having 5-7 full-grown leaves were transferred to an inoculation room and grown at 28°C. All the experiments were performed according to the experiment security regulations of Huazhong Agricultural University (HZAU) and approved by the biosafety committee in HZAU.

*Ralstonia solanacearum* strains were incubated on BG medium [10 g/L peptone, 2.5 g/L glucose, and 1 g/L casamino acids] and BGT medium [BG, 50 mg/L triphenyltetrazolium chloride (TTC), and 15 g/L agar], 200 revolutions per minute overnight according to Tan (Tan et al., 2019). All strains used in this study are listed in **Table S1** (see supporting information). The HZAU091 transformed strains were created by 2.5KV, 6.0 ms electroporation, which introduced the plasmids containing the candidate effectors with their native promoters into the HZAU091 competent cells. Two days later, the clones were tested and confirmed by polymerase chain reaction.

### Genome sequencing of HZAU091 and prediction methods of T3Es in *R. solanacearum*

The genome sequencing was performed by combined technologies including PacBio RS II and Illumina HiSeq2000. The single molecule real-time (SMRT) sequencing was performed on the PacBio RS II platform with a 20 kb library. A paired-end library with an insert size of 400 bp was sequenced using an Illumina HiSeq2000 using PE150 strategy.

The platform and bioinformatics methods were listed in **Table S2** (see supporting information). Since “Ralsto T3E” was the website specially developed for *R. solanacearum* strains, it was chosen for the main prediction. Three other prediction software platforms MAUVE, BRIG-0.95 (BLAST options is -evalue 1e^-5^), FastANI (fastANI -q genome1.fa -r genome2.fa -o output.txt -FragLen), and the representative genome GMI1000 were utilized to predict and rectify the prediction result of the T3Es in HA4-1 and HZAU091. Each predicted T3E gene of HZAU091 was then amplified by polymerase chain reaction and confirmed or corrected by sanger sequencing.

### Pathogenicity analysis

After the ALB28-1 seedlings experienced 2 days of adaptation to the higher temperature, ALB28-1 wild potato plants were inoculated with fresh *R. solanacearum* strain suspensions at OD_600 =_ 0.1 (≈1 × 10^8^ CFU/mL). For the inoculation assay, 10 mL of prepared bacterial suspension was added into the soil matrix around the roots, cutting with a sterile knife beside the stem (about 4cm) to the 5cm depth. Negative controls were roots wounded as above mentioned and inoculated with sterile water. These pathogenicity assays were repeated at least three times in a 28°C inoculation room in HZAU.

The virulence of the strains was evaluated using the Disease Index (DI) and Area under Disease Progress Curve (AUDPC) according to Wang (Wang et al., 2017). The disease index of each plant was then recorded every 2 days consecutively for 3 weeks after infection, using the scales as previously reported (Tan et al., 2019). More than six plants from each potato line were inoculated. The significance treatments of DI values were identified using the method of student’s *t* test by GraphPad Prism 9.0.0. AUDPC values were estimated by SPSS 22.0. Means were compared through student’s *t* test (*p* = 0.05).

For the virulence/hypersensitive response assay of the strains on wild potato leaves, fresh *R. solanacearum* strain suspensions at OD600 = 0.1 (≈1 × 10^8^CFU/mL) were infiltrated into 12 well-expanded 4 weeks-old ALB28-1 leaves.

### Colony morphological observation and growth curve measurement of the *R. solanacearum* strains

The morphology of the bacterial clones used in this study (**Table S1**) was determined using bacterial suspension in the exponential phase, which was diluted, spread onto the TTC medium, and then incubated at 28 °C overnight. To measure the growth curve, 20 mL of BG liquid medium with the bacterial suspension at the starting OD_600 =_ 0.05 in a conical flask was cultured under shaking conditions at 200 rpm and 28°C. Every two hours, 100 μL of bacterial growing culture was pipetted out and added to 900 μL BG liquid to measure the absorbance value at 600 nm. This experiment was repeated four times.

### cAMP concentration assay

The adenylate cyclase assay was performed as previously described with minor modifications (Mukaihara et al., 2010; Zheng et al., 2019). Five μL of bacterial culture at 1×10^8^CFU/mL density of strain HA4-1 and derivatives harboring plasmids were infiltrated into the cutting surface of potato tubers freshly harvested from the field at Huazhong Agriculture University. The tissues were sampled after 28 days of injection. cAMP levels were monitored with a cAMP enzyme immunoassay kit 523 (New east biosciences, PA, USA) according to the instruction.

### Mutagenesis and complementation of *R. solanacearum*

The mutagenesis of effectors in HA4-1 strain was performed as previously described (Cheng et al., 2021). To generate a mutant of the *R. solanacearum* target effector gene, the effector gene was replaced with a cassette harboring spectinomycin resistance. The genome fragment containing the target gene and its two flanking regions was amplified from the strain HA4-1 genome and then was cloned into pCE2-TA-Blunt-Zero vector (Vazyme 5min™ TA/Blunt-Zero Cloning Kit C601-01/02). A reverse amplification of the above recombinant vector was performed to delete the target gene and generate a linear vector only with the flanking region of the effector. A spectinomycin resistance gene (Spe) was connected with the linear to replace the target gene. The mutant plasmid was successfully constructed. Then the spectinomycin cassette with the effector flanking regions was generated, which was used to be transformed into *R. solanacearum* competent cells to replace the target effector gene.

To generate the effector complementation strain, the coding sequences with their native promoters and a Km^r^ cassette were cloned into the plasmid pH7C and then the fragments containing the coding sequences with the effector promoters and the antibiotic gene were amplified and inserted into a permissive chromosome site by transferred into the mutant competent cell, as described previously (Monteiro et al., 2012).

## Results

### HA4-1 and HZAU091 were closely-related and exhibited different virulence levels

**Figure 1:**
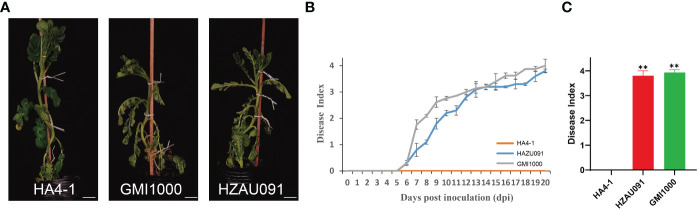
*Ralstonia solanacearum* strains HA4-1, HZAU091, and GMI1000 exhibit significantly different disease progression in the wild potato *Solanum albicans* 28-1 (ALB28-1). **(A)** Photos of ALB28-1 plants 12 days after inoculation with HA4-1, HZAU091, and GMI1000. ALB28-1 inoculated with HA4-1 grew healthy, while plants inoculated with either GMI1000 or HZAU091 showed wilting symptoms. The white scale bar indicates 5 cm. **(B, C)** The disease index of ALB28-1 inoculated with HZAU091, GMI1000, and the avirulent control HA4-1. The concentration of the bacterial suspension was 1×10^8^ CFU/mL. **(C)** The disease index of HZAU091, GMI1000, and HA4-1 were quantified at 20 days post inoculation. The asterisks indicate significant differences between virulent strains and the wild-type HA4-1 (Student’s *t* test, ^**^ *p* < 0.01). All data are presented as mean ± SD for three replicates. The DI values of healthy plants were presented with zero value.

The phylotype I strain of HA4-1 has been identified as an avirulent strain to *Solanum albicans* 28-1 (ALB28-1) which showed a close relationship to the strain HZAU091 (Wang et al., 2017; Tan et al., 2019). To test the resistance of ALB28-1 to HZAU091, we performed the inoculation parallel to HA4-1 and GMI1000. As shown in **Figure 1A**, ALB28-1 inoculated with HA4-1 grew healthy, while plants inoculated with either HZAU091 or GMI1000 showed wilting symptoms after 12 days of inoculation (**Figure 1A**). The disease index also illustrated that HZAU091 was a virulent strain to ALB28-1 (**Figure 1B**). The disease index of the ALB28-1 after 20 days of the inoculation indicated that ALB28-1 was susceptible to HZAU091 and GMI1000 while resistant to HA4-1 (**Figure 1C**). Considering that HZAU091 has a closer genetic relationship with HA4-1 than GMI1000, HZAU091 was selected for an in-depth comparison with HA4-1 to explore the avirulence factors for wild potato. To reveal the mechanism triggering potato immunity, leaves of ALB28-1 plants were inoculated with *R. solanacearum* strains HA4-1 and HZAU091, and resulted in a hypersensitive response (HR) reaction with the former one, while not with the latter. When *hrpB* gene, the core component of the type III secretion system of HA4-1 was mutated, HR was no longer triggered (**Figure S1**). These results also indicated that resistance of ALB28-1 caused by HA4-1 inoculation may be mediated by HA4-1 effector proteins.

**Figure 2:**
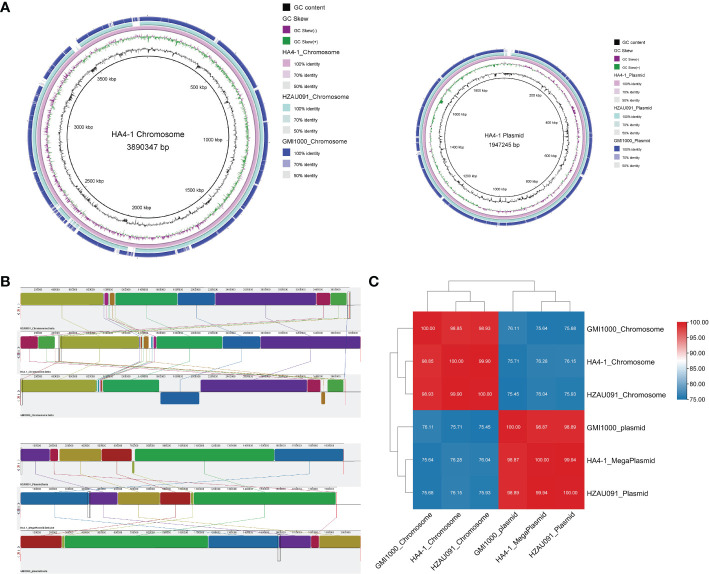
Comparative genomics analyses reveal higher similarity of HA4-1 to HZAU091 than to GMI1000. Genome comparisons between HA4-1, HZAU091, and GMI1000 according to the bioinformatics tools BRIG **(A)**, MAUVE **(B)**, and FastANI **(C)**. **(A)** HZAU091 presented a better alignment with HA4-1 than GMI1000 with HA4-1. The circles from inside to the outside represent GC content, GC Skew, HA4-1, HZAU091, and GMI1000. Left, the chromosome comparison among the three genomes. Right, the plasmid comparison among the three genomes. **(B)** Chromosome comparison is shown above, plasmid comparison is shown down. Upper sequence, HZAU091. Medium sequence, HA4-1. Lower sequence, GMI1000. Boxes with the same color indicate the syntenic regions. Boxes below the horizontal line indicate inverted regions. **(C)** The ANI analysis result of HA4-1, HZAU091, and GMI1000.

The genome of HZAU091 was sequenced and deposited at GenBank under BioSample and Bioproject accession number SAMN28868074 and PRJNA846088, respectively. HZAU091 was composed of a circular chromosome and a megaplasmid. The whole-genome size of HZAU091 was 5,757,034 bp. A total of 5,092 CDS (coding sequence) genes were predicted. Compared to HA4-1, the genome size of HZAU091 was smaller (**Table S3**, see supporting information). HA4-1 had been proven previously to have a closer relationship with HZAU091 than GMI1000 based on the alignment of the partial endoglucanase (*egl*) sequence (Wang et al., 2017). However, the similarity of these three strains on the genome level remained unclear. To carry out the alignment, Mauve, BRIG, and FastANI were applied (Alikhan et al., 2011; Jain et al., 2018). The collinearity analysis indicated that HZAU091 had fewer gaps in both chromosome and plasmid levels than GMI1000. Apart from the gaps, HZAU091 didn’t possess the two inverted regions as GMI1000 possessed (**Figures 2A, B**). Average Nucleotide Identity (ANI) is an indicator of the comparison of two genomic similarities at nucleotide levels. If the ANI of organisms remarkably presents ≥95%, they belong to the same species (Konstantinidis and Tiedje, 2005). The ANI analysis of the chromosome and plasmid among the three genomes showed that the HA4-1 chromosome sequence had 99.90% nucleotide identity with HZAU091 chromosome while with only 98.85% nucleotide identity with GMI1000. As for megaplasmid comparison, HA4-1 had 99.94% nucleotide identity with HZAU091 while 98.87% with GMI1000 (**Figure 2C**). Thus, HZAU091 presented a more closely related relationship with HA4-1 than GMI1000 at the genome level.

### Comparison of T3Es between HA4-1 and HZAU091 identified seven distinct effectors

**Figure 3:**
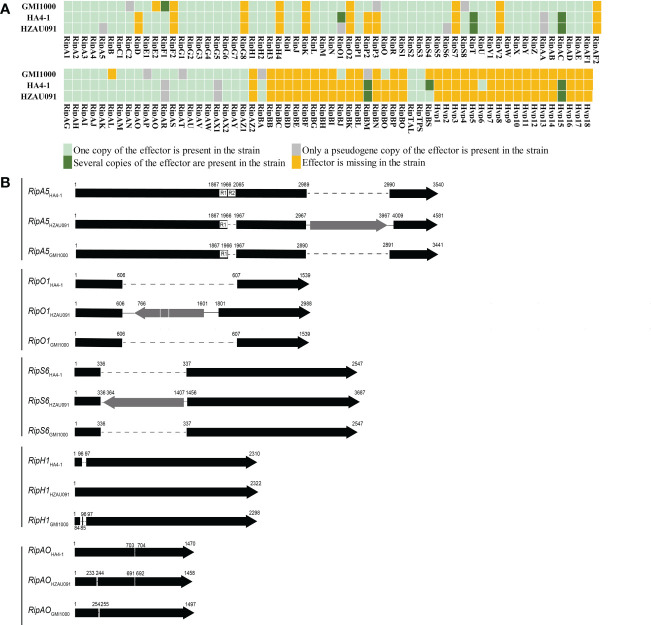
HA4-1 and HZAU091 exhibit differences in their T3Es repertoires. **(A)** Distributions of repertoires of T3Es in HA4-1 and HZAU091. GMI1000 is a representative T3Es repertoire. Different colors of the boxes indicate different copy numbers in the genome. Light green boxes indicate the effector is present in the strain with only one copy. Dark green boxes indicate several copies of the effector are present in the strain. Grey boxes indicate pseudo-effectors in the strain. Yellow boxes indicate the effector is missing in the strain. Five effectors RipA5, RipO1, RipS6, RipBS, and Hyp6 have different copy numbers in HA4-1 and HZAU091. **(B)** Sketch structure maps of T3Es different between HA4-1 and HZAU091. Grey arrows indicate the insertion. Dotted lines indicate the absence of a region in the gene. RipA5, RipO1, and RipS6 in HZAU091 exhibit large fragment insertions than HA4-1. RipH1 and RipAO in HZAU091 exhibit 12 base pair insertion and deletion.

With the genomes of an avirulent strain and a virulent strain, we used comparative genomics to identify the core effectors. The first step was to identify the T3Es in these two strains. Four prediction software platforms and a representative genome were utilized to predict and rectify the prediction result of the T3Es in HA4-1 and HZAU091. Since “Ralsto T3E” was the website specially developed for *R. Solanacearum* strains, it was chosen for the main prediction (**Table S2**). The result showed that HA4-1, HZAU091, and GMI1000 possessed 77, 74, and 78 effectors, respectively (**Figure 3A**). Of them, 71 effectors coexisted in all three genomes. No unique effectors were identified for HZAU091. Each predicted T3E gene of HZAU091 was amplified using polymerase chain reaction and confirmed or corrected by Sanger sequencing. All effectors of HZAU091 were present in the HA4-1 genome. Correspondingly, HA4-1 owned three effectors absent in HZAU091.

Since Hyp6 presented only in HA4-1, it was chosen as a candidate avirulence effector first. Both HA4-1 and HZAU091 had several genes with multiple copies or pseudogenes in the genome. RipO1 and RipBS were chosen among the three genomes based on the different copy numbers in HA4-1 and HZAU091. RipO1 was predicted to have two copies in HA4-1 genome, one copy CFM90_21005 was identical to RipO1^GMI1000^. Compared with CFM90_21005, RipO1^HZAU091^ had an 1194 bp insertion fragment containing a transposase. We judged the other one CFM90_10880 to be a pseudogene since this copy was <5% similar to RipO1^GMI1000^, it possessed many mismatches and gaps. Thus, we choose the first copy CFM90_21005 to conduct the following virulence/HR assay of RipO1. RipBS, which possessed three copies in HA4-1, was absent in GMI1000 and presented only identical copy in HZAU091. RipBS had three identical copies in HA4-1 genome positioned in CFM90_18115, CFM90_26330, and CFM90_26510. RipBS was absence in GMI1000, and possessed only one identical copy gene2801 in HZAU091. These three copies were surrounded by the transposases, mobile element proteins, and hypothetical proteins. RipA5, RipO1, and RipS6 both had a transposon in the gene CDS and were identified to be pseudogenes in HZAU091 while functional in HA4-1 (**Figure 3B**). Apart from the insertion of the transposon, RipA5 also had a 99 bp deletion in HZAU091. The DNA sequences of common effectors in HZAU091 and HA4-1 were aligned, then the difference between RipH1 and RipAO was detected. In summary, we identified seven distinct effectors as candidates. The basic information of the seven candidates was listed in **Table S4** (see supporting information).

### Several candidate effectors introduced into HZAU091 could boost the immunity of ALB28-1

**Figure 4:**
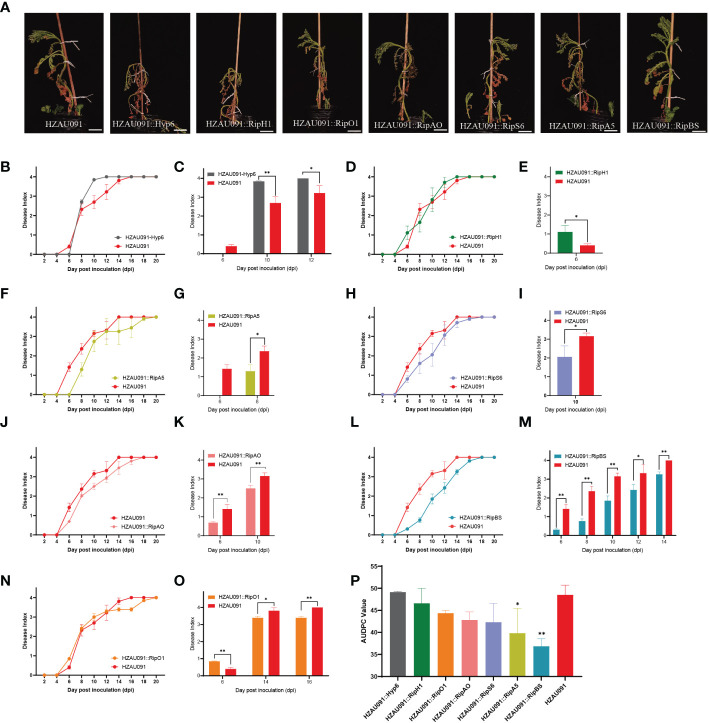
HZAU091 virulence is compromised in different degrees when carrying each of the seven candidate effector proteins. **(A)** The phenotypes of *Solanum albicans* 28-1 (ALB28-1) at 12 dpi. The white scale bar indicates 10 cm. The concentration of the bacterial suspension was 1×10^8^ CFU/mL. **(B, D, F, H, J, L, N)** The disease index of ALB28-1 inoculated with the virulent strain HZAU091 carrying effectors from HA4-1 for 20 days. **(C, E, G, I, K, M, O)** The corresponding significant differences of DI were compared between the material ALB28-1 inoculated with HZAU091 carrying each of the seven candidate effector proteins and the virulent control HZAU091 at depicted time points. **(P)** The area under the disease progress curve (AUDPC) value of pathogenicity of eight strains. The virulence of HZAU091 carrying effectors in HA4-1 was compared with HZAU091. Data are presented as mean ± SD for three representative experimental replicates. Asterisks indicate significant differences of results (Student’s *t* test, ^*^ *p* < 0.05, ^**^ *p* < 0.01). The DI values of healthy plants were presented with zero value. The experiments were repeated at least three times with similar results.

Virulence assays were carried out with seven candidate effectors from HA4-1 which were driven under their native promoters and then transferred into the virulent strain HZAU091. As shown in **Figure 4A**, the virulence of HZAU091 carrying the candidate effectors was offset to different degrees. ALB28-1 exhibited better resistance against HZAU091::RipBS than wild-type HZAU091 and other virulent strains of HZAU091 carrying effectors from HA4-1 after 12 days of inoculation. To evaluate the ability of each candidate effector to reduce the virulence of HZAU091, all DI progression curves had been shown in **Figures 4B–O**. With HZAU091::Hyp6 inoculation, a higher disease index was observed than HZAU091 (**Figure 4C**). As for HZAU091::RipH1, the development trend showed a trade-off with HZAU091 (**Figure 4D**). ALB28-1 exhibited more susceptibility against *R. solanacearum* strain HZAU091 carrying RipH1 at the early stage (**Figure 4E**). HZAU091::RipA5, HZAU091::RipS6, and HZAU091::RipAO significantly depressed the virulence at the early stage (**Figures 4F–K**). HZAU091::RipBS significantly reduced the virulence of HZAU091 throughout the inoculation progression (**Figures 4L, M**). HZAU091::RipO1 significantly reduced the virulence at the late stage (**Figures 4N, O**). The significant difference analysis of HAZU091 carrying candidate effectors with wild-type HZAU091 was shown beside the corresponding disease index curve. Apart from the disease index, the AUDPC assay should also be taken into consideration to assess the overall pathogenicity of the strains. According to the area under the disease progress curve (AUDPC) caused by the eight strains (**Figure 4P**) and the above DI results, we demonstrated that the introduction of RipBS and RipA5 could significantly reduce the pathogenicity of HZAU091.

**Figure 5:**
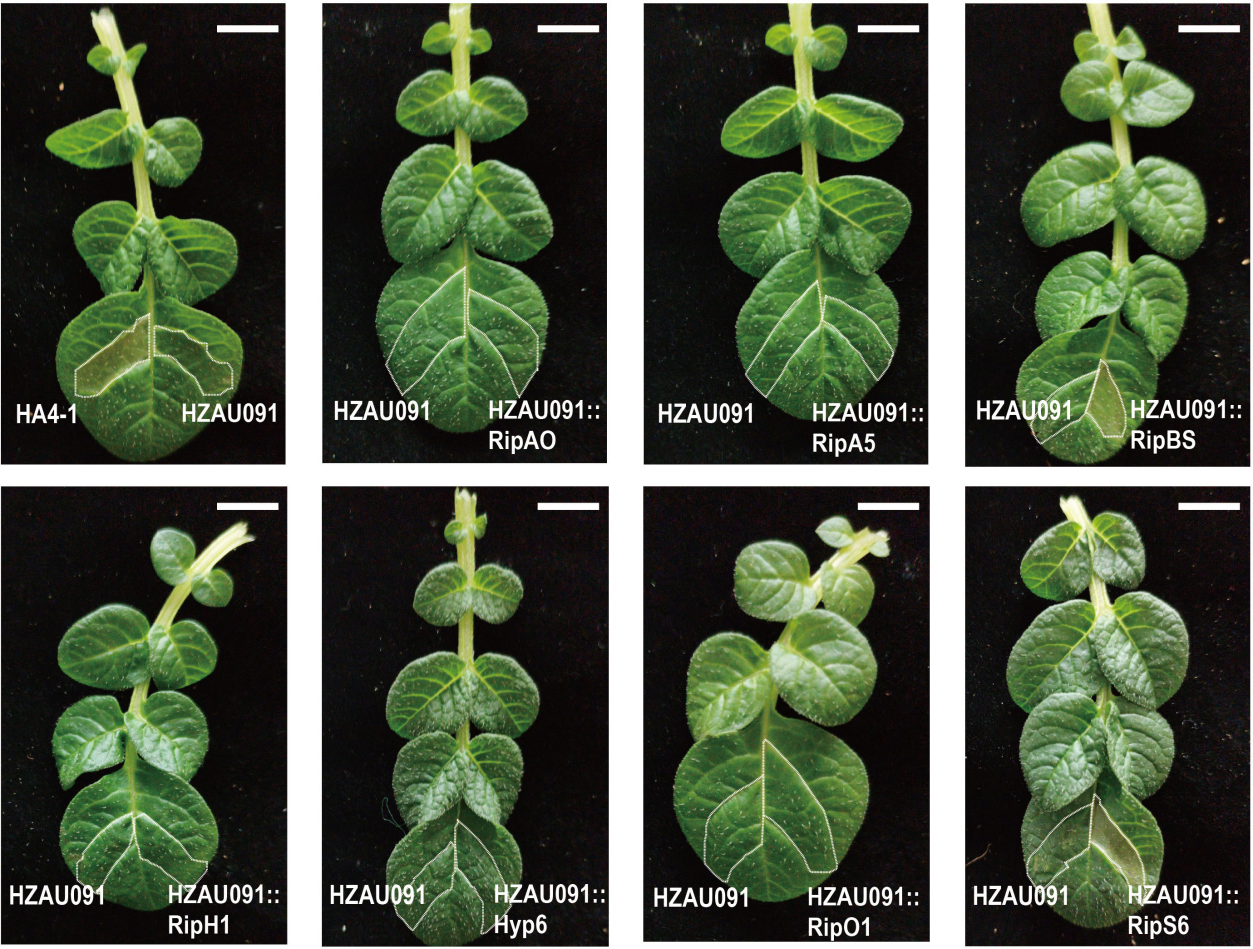
HZAU091 carrying RipBS and RipS6 elicited the HR of ALB28-1 leaves. All candidate effectors of HA4-1 were introduced into HZAU091. HA4-1 (avirulent) could cause HR in ALB28-1 leaves while HZAU091 (virulent) failed. HA4-1 and HZAU091 were wild type controls. HZAU091::RipBS and HZAU091::RipS6 could elicit the HR as HA4-1. All bacterial strains were resuspended at OD_600 =_ 0.1 (≈1 × 10^8^CFU/mL) and infiltrated into 12 ALB28-1 leave replicates. The experiments were repeated at least three times with similar results. The white scale bar indicates 1 cm. The photo was taken at 3 dpi.

Even though no candidate effectors with the ability to induce complete loss of pathogenicity were discovered, the result of the experiment to evaluate the virulence of HZAU091 carrying effectors from HA4-1 still yielded useful information. The delivery of RipBS and RipA5 by HZAU091 significantly reduced the virulence. ALB28-1 leaves infiltrated with HA4-1 caused HR while HA4-1 ΔhrpB and HZAU091 failed. To investigate whether the HR was related to these candidate effectors, all chosen effectors introduced into HZAU091 were infiltrated into ALB28-1 leaves. Except for HZAU091::RipS6 and HZAU091::RipBS triggering cell death, the ALB28-1 phenotypes infiltrated with other HZAU091 complementation strains were consistent with HZAU091 (**Figure 5**). RipBS and RipS6 were recognized by ALB28-1 during the infiltration in the leaf.

### Mutation of candidate effectors confers pathogenicity on ALB28-1

**Figure 6:**
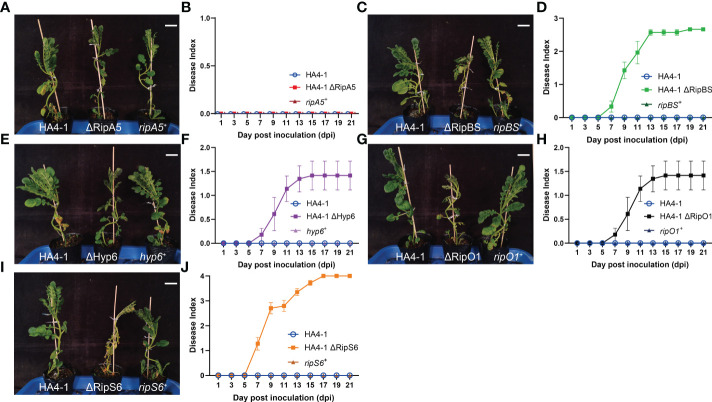
Mutation in predicted T3Es increased virulence of HA4-1. **(A, C, E, G, I)** The phenotypes of ALB28-1 inoculated with HA4-1, HA4-1 mutants and their complementation strains at 15 dpi. White scale bar indicates 5 cm. ALB28-1 was resistant to HA4-1, HA4-1 ΔRipA5 and all complementation strains, while susceptible to other HA4-1 mutants. **(B, D, F, H, J)** The disease index of ALB28-1 inoculated with HA4-1, HA4-1 effector mutants and their complementation strains within 21 days. The concentration of the bacterial suspension was 1×10^8^ CFU/mL. Error bars represent mean ± SD for three representative experimental replicates. The DI values of healthy plants were presented with zero value. The experiments were repeated at least three times with similar results.

Virulence assays to evaluate the role of T3Es include different approaches. Compared to the above experiment to evaluate the virulence of HZAU091 carrying effectors from HA4-1, gene mutation and complementation was a more classical way to evaluate the virulence. Therefore, we constructed the mutants by substituting the entire CDS region of the predicted effectors into the CDS region of antibiotic genes in HA4-1 strain genome. It should be clarified that in order to rigorously verify the virulence function of RipBS, the ΔRipBS mutant used in the following experiments is the triple mutant of three copy numbers of RipBS (**Figure S2**). The mutants exhibit the same morphology and growth curves as the wild type strains, which illustrated that the pathogenicity difference was mainly due to the absence of the candidate effectors (**Figure S3**). Then the virulence of successfully mutated strains (ΔRipA5, ΔRipBS, ΔHyp6, ΔRipS6, and ΔRipO1) and their complementary strains was tested (**Figures 6A–J**). After 12 days of mutated strains’ inoculation, all plants showed wilting symptoms except for that inoculated by ΔRipA5 and HA4-1 which grew healthy (**Figure 6A**). All plants inoculated with complementation strains showed resistance at the same time (**Figures 6A, C, E, G, I**). When RipS6 was mutated, the wilt symptom of ALB28-1 was the most severe (**Figure 6I**), and the pathogenic process was the most rapid during the invasion (**Figure 6J**). The DI of the mutant strains was ΔRipS6 > ΔRipBS > ΔRipO1 > ΔHyp6 > ΔRipA5 = HA4-1 (**Figures 6B, D, F, H, J**). All these mutated strains were infiltrated into ALB28-1 leaves to verify the core avirulence effector as the complementation strains, but none of them resulted in the disappearance of the HR (**Figure S4**), which might be as a result of the effector functional redundancy.

Taken together, with the combination of the pathogenicity results of the virulent strain HZAU091 carrying effectors from HA4-1 and the mutant strains and its complementary strains, RipBS and RipS6 were regarded as key avirulence effectors that could be recognized by ALB28-1. Apart from the above experiments conducted using the resistance host ALB28-1, the host-specific recognition was then confirmed by using an HA4-1 susceptible host *Solanum tuberosum L*. cultivar E3 (**Figure S5**). E3, inoculated with HA4-1, HA4-1 ΔRipBS, and HA4-1 ΔRipS6, showed wilting symptoms with no significant difference (**Figure S5A**). *R. s* strains HZAU091 carrying RipS6 and RipBS also conferred virulence to E3 with no significant difference (**Figure S5B**). Future work is needed to gain further understanding of the molecular function of RipBS and RipS6.

### RipBS is a novel T3E unique in *R. solanacearum* strains

**Figure 7:**
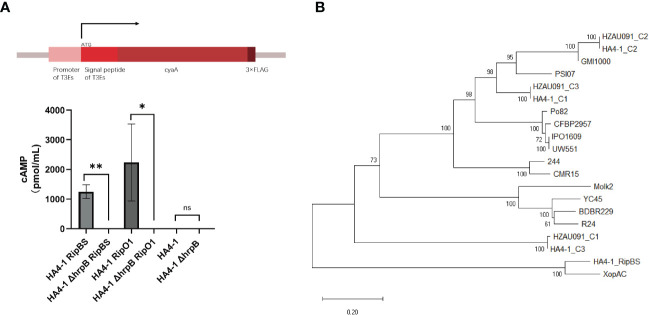
RipBS was identified as a novel effector. **(A)** The Cya reporter system to identify the T3Es based on the gene expression study method. HA4-1 ΔhrpB was a core T3SS system mutated strain that lost the ability to deliver the T3Es into the plant cells. HA4-1 ΔhrpB strains acted as controls. Error bars represent mean ± SD for three biological replicates. Asterisks indicate significant differences (Student’s *t* test, *p < 0.05, **p <0.01). ns means no significant difference. The DI values of healthy plants were presented with zero value. **(B)** The phylogenetic tree of RipBS in all *R. solanacearum* strains containing RipAC (C1, C2, C3 indicated different copies of RipAC in either HA4-1 or HZAU091).

Since RipBS was predicted as T3Es only by the bioinformatic method (Tan et al., 2019), this study experimentally supplemented the demonstration utilizing the Cya reporter system. In this system, the CyaA protein could be delivered into the plant cell when T3SS perceived the signal peptide of the predicted effectors (Mukaihara et al., 2010). If the core element of T3SS was abolished, the T3Es would lose the ability to be delivered into the host plant cells to trigger or suppress the plant immunity. Therefore, *hrpB*, encoding the structure component of theT3SS, was mutated and used as a negative control. Since RipO1 was previously discovered in other strains as a definite T3E (Peeters et al., 2013), we used it as a positive control. Then all recombinant plasmids, carrying both effector gene and the *cyaA* gene, were introduced into the HA4-1 and HA4-1 ΔhrpB, and all these *R. solanacearum* strains were infiltrated into the potato tubers for 28 days in dark. With the successful delivery of the fusion protein RipBS^SP^-Cya and RipO1^SP^-Cya to the host plant cell, Cya protein was perceived by plants and robustly elevated the concentration of cAMP due to the cascade reactions. When the *hrpB* of HA4-1 was mutated, no significant rise in concentration was observed in any experimental groups (**Figure 7A**). This result proved that RipBS was secreted through the type III secretion system and was a novel T3E in the *R. solanacearum*.

In this experiment, when the T3Es prediction of HA4-1 was initially made, RipBS was not within the range of the prediction results. However, RipBS was originally predicted using bioinformatics methods as a new T3E (Tan et al., 2019). Therefore, it was speculated that RipBS can be classified in this experiment as some other effector proteins. The sequence of RipBS was then compared with the effector protein with multiple copies in HA4-1. It was found that RipBS was classified into the RipAC multi-copy sequences of HA4-1. As shown in a phylogenetic tree of RipAC sequences among all sequenced *R. solanacearum* strains, HA4-1 presented at least three RipAC copy numbers (**Figure 7B**). The nucleotide sequence of *RipBS* was identical to the unique copy from HA4-1 RipAC. Then the amino acid sequence of this unique RipAC copy was also blasted in UniproKB and the result showed the alignment with *Xanthomonas campestris* pv. *campestris* XopAC as RipBS alignment (**Figure S6**). In summary, the T3Es prediction result was rectified. That is, RipBS was identified as a novel T3E rather than a copy of RipAC.

## Discussion

This work determined the pathogenicity of HA4-1 and HZAU091 to a wild potato accession ALB28-1. ALB28-1 was resistant to HA4-1 while susceptible to HZAU091 (**Figures 1A, B**). The genome sequence of HZAU091 was sequenced and the comparative genomics analysis confirmed that HZAU091 was more closely related to HA4-1 on the genome level (**Figure 2**). Using the comparative genomics method, seven distinct effectors were predicted in HA4-1, among which Hyp6 was found to present only in HA4-1 compared with HZAU091 (**Figure 3A**). The virulence assays conducted with HZAU091 harboring HA4-1 effectors and with T3E mutated HA4-1 clones, demonstrated that certain predicted effectors have a strong role in conferring avirulence to *R. solanacearum* in the wild potato. In particular, our study revealed that RipS6 and RipBS, a novel predicted effector, substantially contribute to the resistance of ALB28-1 against *R. solanacearum* (**Figures 4A**, **6I**).

RipBS was first predicted as a novel effector in the *R. solanacearum* (Tan et al., 2019), but was not identified by the gene expression experiment. In this work, RipBS was predicted to possess an *hrpII* box (**Table S4**) and was confirmed as a T3E based on the cAMP experiment using the Cya reporter system (**Figure 7A**). The amino acid alignment of RipBS with RipAC homologs and XopAC revealed that RipBS had a closer relationship with the *Xanthomonas campestris* pv. *Campestris* (Xcc) effector XopAC than with the *Ralstonia solanacearum* effector RipAC (**Figure 7B**). XopAC is also named AvrAC due to its avirulence ability. XopAC can trigger the immunity of Arabidopsis ecotype Col-0 relying on the LRR (Leucine-rich repeat)and fic domains (Xu et al., 2008; Guy et al., 2013). However, AvrAC has also been reported as a virulence factor that can suppress plant immunity by specifically targeting the plant immune kinases BIK1 and RIPK1 with its uridine 5’-monophosphate transferase activity and inhibiting flg22-induced MAPK activation by targeting the RLCKVII (Feng et al., 2012; Yamaguchi et al., 2013). By contrast, the host developed decoy substrate PBL2 and pseudokinase RKS1 as well as ZAR1 to recognize and consume AvrAC to make a successful intercept for BIK1 and trigger the plant immunity (Wang et al., 2015). When RipBS was mutated, the pathogenicity of the RipBS-mutant strain showed an obvious increase (**Figures 6C, D**), illustrating that plants may lose the target effector to recognize and trigger immunity. Thus, RipBS was demonstrated to be an avirulence effector in HA4-1. The mechanism of how RipBS confer avirulence remained to be discovered. Since RipBS also possessed the LRR and fic domains as XopAC, we hypothesized that these domains also contribute to its biological function.

RipS6 may act as an avirulence effector as its mutation led to strong virulence to HA4-1 in ALB28-1. It is worth noting that in our findings, RipS6 was also present in the GMI1000 genome (**Figure 3A**), while the inoculation result indicated that GMI1000 were virulent to ALB28-1 (**Figure 1A**). For effector protein prediction, we amplified all predicted protein gene fragments using polymerase chain reactions and sequenced them, and compared with the GMI1000 reference effectors. RipS6^GMI1000^ has multiple missense mutations at the nucleotide level compared with RipS6^HA4-1^ (**Table S4**). Therefore, it is speculated that the missense mutations in GMI1000 may alter the biological function of RipS6, and that ALB28-1 may not recognize RipS6^GMI1000^ and trigger the plant immunity. RipS6 belongs to the RipS (SKWP) family, which contains 12–18 tandem repeats of a novel 42aa motif (Mukaihara and Tamura, 2009). In this effector family, RipS1 has been reported to suppress the flg22-induced ROS (Landry et al., 2020). RipS4 (previously named RSc1839) can contribute to bacterial fitness (Macho et al., 2010). The RipS multiprotein of OE1-1, a Japanese *R. solanacearum* strain, has been proven to collectively contribute to bacterial virulence to eggplant. In the meantime, RipS1, RipS4, and RipS5 are the major RipS effectors for disease development (Chen et al., 2021). However, few mechanisms of how the SKWP family modulates plant immunity have been reported. Our study first proved that the effector RipS6 reduce bacterial virulence to potato.

The inoculation results of HA4-1 mutant strains showed that RipBS, RipS6, RipO1, and Hyp6 all contribute to the avirulence to ALB28-1, while only RipBS and RipS6 could reduce the virulence of HZAU091 according to the inoculation of the virulent strain HZAU091 carrying these two effectors in HA4-1. The leaf inoculation results also showed that HZAU091::RipS6 and HZAU091::RipBS are the only effectors that could still cause HR as HA4-1 (**Figure 5**). The introduction of RipS6, RipO1, and Hyp6 failed to significantly reduce the plant susceptibility caused by HZAU091 infection. In many cases, some effectors could inhibit the ETI (Effector-Triggered Immunity) triggered by another effector or ETI-associated components, for example, RipAY could inhibit the ETI triggered by RipE1 (Sang et al., 2020), XopQ inhibits cell death triggered by ETI-associated MAP kinase cascade MAPKKKα MEK2/SIPK and by several *R/avr* gene pairs (Teper et al., 2014). It was speculated that some effectors inside HZAU091 may also inhibit the ETI elicited by RipO1 and Hyp6 of HA4-1. To confirm this hypothesis and uncover the effector inhibitor, RipS6, RipO1, and Hyp6 of HA4-1 can be complemented into the HZAU091 mutant strain of a certain effector and then perform inoculation experiments in future studies.

The leaf inoculation results showed that single effector mutated strains could still cause HR as HA4-1 (**Figure S4**). Effector functional redundancy may explain this result, for example, RipB, RipAA, and RipP1 contribute in part to the avirulence of RS1000 (Nakano and Mukaihara, 2019). Several effector proteins in HA4-1 may be recognized by ALB28-1 leaves to trigger the immune response. To investigate whether the HR was related to these candidate effectors, introducing the candidate effector into HAZU091. All chosen effectors introduced into HZAU091 were infiltrated into ALB28-1 leaves. HZAU091::RipS6 and HZAU091::RipBS elicited the HR which HZAU091 and other HZAU091 complementation strains failed (**Figure 5**). To uncover the effector function in the future, RipBS/RipS6 double mutant strains and even multiple mutant strains should be constructed.

Our research results identified the effectors from HA4-1 that could boost plant immunity, conforming that comparative genomics and T3Es repertoires analysis of different virulence strains was a useful way to identify core avirulence effectors for the plants that lack well-known resistance resources. Based on comparative genomics and pathogenicity assays of two strains under the same genetic background, researchers can shed more light on these areas. The results of this research could be beneficial to promoting the study of the interaction mechanism between potato and *R. solanacearum*, and further uncovering the resistance gene to bacterial wilt. However, only one wild potato accession was used in this study. Researchers still face significant challenges in advancing the development of commercial potato bacterial wilt resistance breeding.

## Data availability statement

The datasets presented in this study can be found in online repositories. The names of the repository/repositories and accession number(s) can be found in the article/**Supplementary Material**.

## Author contributions

MH and XT conducted the experiments. HC and BS conceived the research and designed the experiments and supervised the molecular biology experiments. YW helped to screen the potato bacterial resistance material. MH wrote the manuscript. BS, XT, HC, BW, and DC guided the paper writing. All authors contributed to the article and approved the submitted version.
