# Supplementary material for: Comparative genomic analysis of Ralstonia solanacearum reveals candidate avirulence effectors in HA4-1 triggering wild potato immunity
Source: Front Plant Sci. 2023 Feb 23;14:1075042. doi: 10.3389/fpls.2023.1075042 (PMC9997847; doi:10.3389/fpls.2023.1075042)

**Table S1. All strains and vectors used in this study.**

| **Strains** | **Description** | **Usage in this study** |
| --- | --- | --- |
| **HA4-1** | ***Solanum albicans* 28-1 is resistant to HA4-1** | **Virulence assay, cAMP assay** |
| **HZAU091** | ***Solanum albicans* 28-1 is susceptible to HZAU091** | **Virulence assay, cAMP assay** |
| **HZAU091::RipA5** | **HZAU091 expressing the effector RipA5 ^HA4-1^** | **Virulence assay** |
| **HZAU091::RipAO** | **HZAU091 expressing the effector RipAO ^HA4-1^** | **Virulence assay** |
| **HZAU091::RipBS** | **HZAU091 expressing the effector RipBS ^HA4-1^** | **Virulence assay** |
| **HZAU091::RipH1** | **HZAU091 expressing the effector RipH1 ^HA4-1^** | **Virulence assay** |
| **HZAU091::RipS6** | **HZAU091 expressing the effector RipS6 ^HA4-1^** | **Virulence assay** |
| **HZAU091::RipO1** | **HZAU091 expressing the effector RipO1 ^HA4-1^** | **Virulence assay** |
| **HZAU091::Hyp6** | **HZAU091 expressing the effector Hyp6 ^HA4-1^** | **Virulence assay** |
| **HA4-1 ΔRipA5** | **The effector RipA5 was mutated in HA4-1** | **Virulence assay** |
| **HA4-1 ΔRipBS** | **The effector RipBS was mutated in HA4-1** | **Virulence assay** |
| **HA4-1 ΔRipS6** | **The effector RipS6 was mutated in HA4-1** | **Virulence assay** |
| **HA4-1 ΔRipO1** | **The effector RipO1 was mutated in HA4-1** | **Virulence assay** |
| **HA4-1 ΔHyp6** | **The hypothetical effector Hyp6 was mutated in HA4-1** | **Virulence assay** |
| **HA4-1 ΔhrpB** | **T3SS core element gene *hrpB* mutated with effectors loss the ability to be delivered into the plant cells** | **cAMP assay** |
| **HA4-1 ΔhrpB RipBS** | **Plasmid cyaA-RipBS expressing cAMP was introduced into the HA4-1 ΔhrpB competent cells** | **cAMP assay** |
| **HA4-1 ΔhrpB RipO1** | **Plasmid cyaA-RipO1 expressing cAMP was introduced into the HA4-1 ΔhrpB competent cells** | **cAMP assay** |

**Table S2. The prediction of T3Es with the use of “Ralsto T3E” and “Effectdior”.** The prediction was applied with the sequenced genome fasta file. The prediction data was rectified with pEffect. The representative genome GMI1000 was utilized to predict and rectify the prediction result of the T3Es in HA4-1 and HZAU091.

| **Methods** | **Website link** | **Published Year** | **Usage in this work** | **References** |
| --- | --- | --- | --- | --- |
| Raslto T3Es | [Ralsto T3E (inra.fr)](https://iant.toulouse.inra.fr/bacteria/annotation/site/prj/T3Ev3/) | 2013 | Prediction | (Peeters et al., 2013) |
| Eff3ctidor | [Eff3ctidor (tau.ac.il)](https://effectidor.tau.ac.il/) | 2022 | Rectification | (Wagner et al., 2022) |
| EffctiveT3 | [EffectiveT3 \| EffectiveDB (univie.ac.at)](https://effectors.csb.univie.ac.at/method/effectivet3) | 2010 | Rectification | (Jehl et al., 2011) |
| pEffect | [pEffect - Type III Effector Proteins Prediction Server (bromberglab.org)](https://services.bromberglab.org/peffect/) | 2016 | Rectification | (Goldberg et al., 2016) |

**Table S3. Comparison of genome information of HA4-1 and HZAU091.**

|  | HA4-1 | HZAU091 | GMI1000 |
| --- | --- | --- | --- |
| **Accession** | GCA_003999715.1 | -- | GCA_000009125.1 |
| **Bioproject** | PRJNA392775 | PRJNA846088 | PRJNA13 |
| **Genome size(bp)** | 5,981,347 | 5,750,034 | 5,810,922 |
| **G + C Ratio (%)** | 66.68 | 66.88 | 66.97 |
| **Predicted no. of CDS** | 4930 | 5092 | 5129 |
| **rRNAs** | 59 | 12 | -- |
| **tRNAs** | 12 | 58 | 58 |
| **Pseudogene(no.)** | 158 | 70 | 129 |

**Table S4. Information of the seven chosen predicted proteins.**

| **HA4-1** | **HA4-1 gene ID** | **Difference in HZAU091** | **Difference in GMI1000** | **Conserved domains** | | ***HrpⅡ* box** |
| --- | --- | --- | --- | --- | --- | --- |
|  |  |  |  | **Accession** | **Name** |  |
| **RipA5** | **CFM90_25415** | **Transposon insertion and 99bp deletion** | **99bp deletion, point mutation S22G, T536A** | **---** | **---** | **Yes** |
| **RipO1** | **CFM90_21005/ CFM90_10880** | **Transposon insertion** | **Single copy** | **---** | **---** | **Yes** |
| **RipS6** | **CFM90_04350** | **Transposon insertion** | **Point mutation C24R, R67P, H78L, A79V, L98F, G101L, G222H, R241G, A487D, S609P, G686H, H771G, K836T** | **cl35774** | **PRK09169 Superfamily** | **---** |
| **RipAO** | **CFM90_18395** | **12bp deletion** | **24 bp insertion, 18bp deletion, point mutation R47K, L274P, T285A** | **---** | **---** | **Yes** |
| **RipH1** | **CFM90_07595** | **12bp insertion** | **12bp deletion, point mutation V39A, G42A, L66S, R164G, G189K, H237G, T238A, T252K, P280S, S293N, T355A, S380A, T478M, N536D, G648V, P699S, A752V, K759R** | **---** | **---** | **---** |
| **RipBS** | **CFM90_18115/**  **CFM90_26510/**  **CFM90_26330** | **Single copy** | **absence** | **pfam02661 /cl00960** | **Fic/DOC family** | **Yes** |
|  |  |  |  | **COG4886**  **/cl34836** | **Leucine-rich repeat (LRR) protein** |  |
| **RipHyp6** | **CFM90_11585** | **Absence** | **absence** | **---** | **---** | **Yes** |

**Figure S1. HA4-1 could trigger the HR of ALB28-1 leaves.** HA4-1 (avirulent) triggered the HR of ALB28-1 leaves. HZAU091(virulent) and HA4-1 ΔhrpB (T3SS core element mutant) failed to elicit the HR. All strains were resuspended at OD_600_=0.1 (≈1 × 10^8^ CFU/mL) and infiltrated into 12 ALB28-1 leaves. The experiments were repeated at least three times with similar results. The white scale bar indicates 1 cm. The photo was taken at 3 dpi.

**
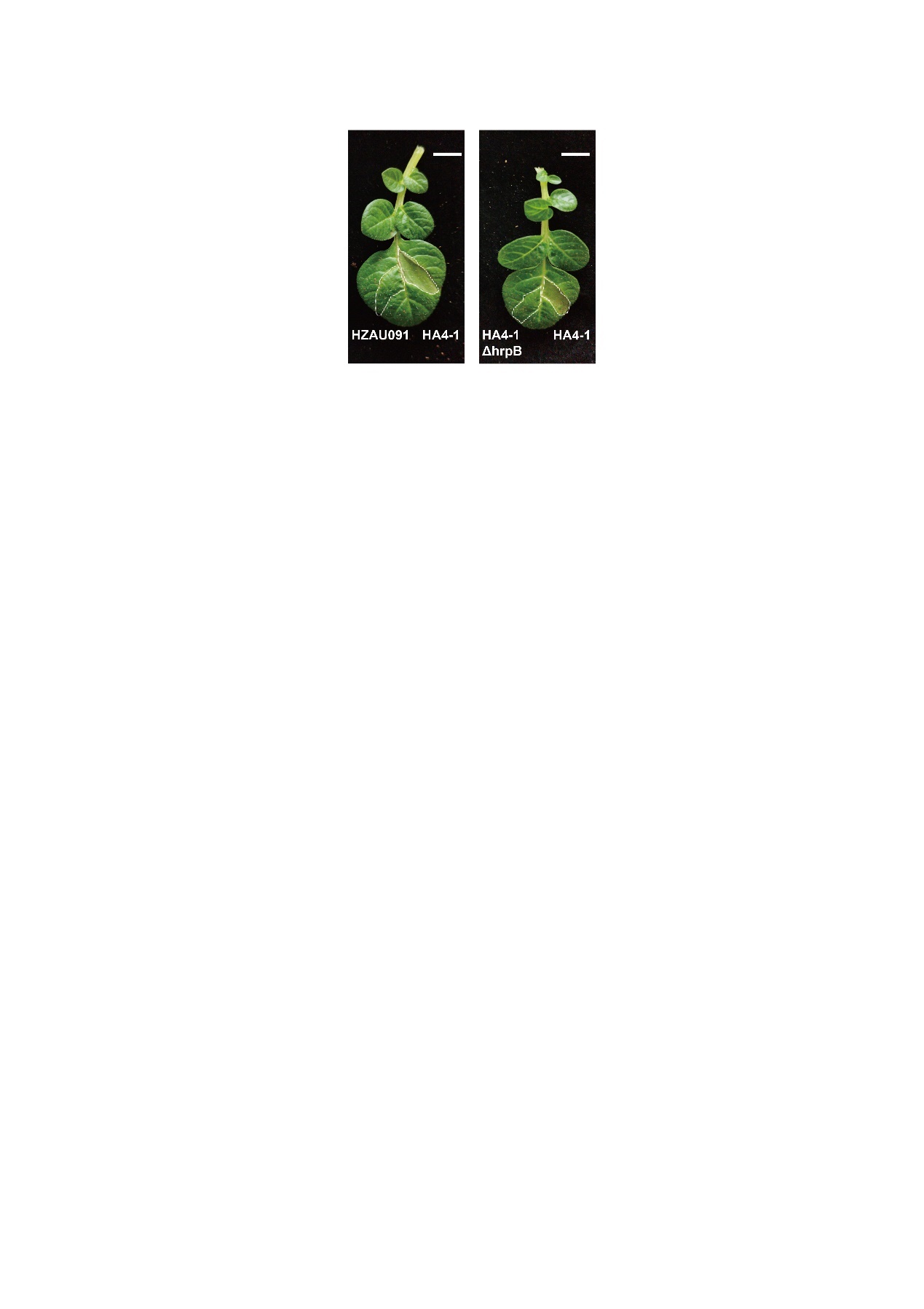
**

**Figure S2 The agarose gel electrophoresis plot of RipBS triple-mutant.** BS1: CFM90_18115, BS2: CFM90_26330, BS1: CFM90_26510. DL2000: the DNA marker. The test fragment of RipBS product is 1284bp. The test fragment of the RipBS-spe product is 1284bp. pCE2-RipBS, pCE2-RipBS were positive controls separately.

**
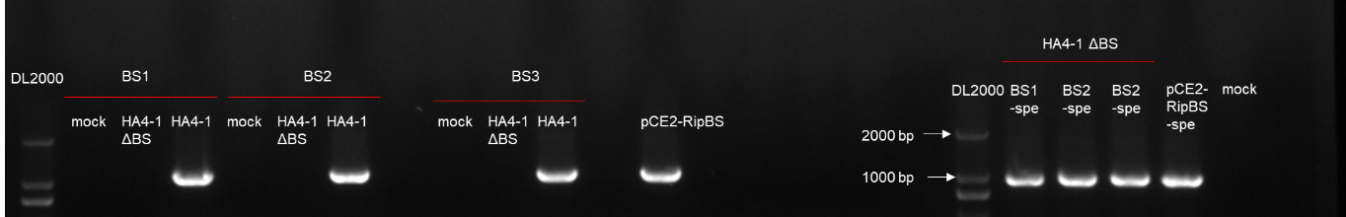
**

**Figure S3 Strains of HA4-1 with mutated T3E genes exhibit normal growth phenotypes. (A) – (F)** Phenotypes of *Ralstonia solanacearum* strains harboring mutations in predicted type three effectors are shown. Red arrows indicate typical colonies with a pink center and milky edge. **(G)** Growth curve of the mutated strains. The growth curve was tested at OD_600_=0.05 at the beginning. The significant differences of bacterial concentrations were compared between the HA4-1 mutant strains with the wild-type HA4-1. Data are presented as mean ± SD for four replicates. No significant difference in bacterial concentrations was observed during the inoculation (Student’s *t* test, ns = no significant difference). 12 h was chosen as a time point that bacterial concentration raised rapidly.


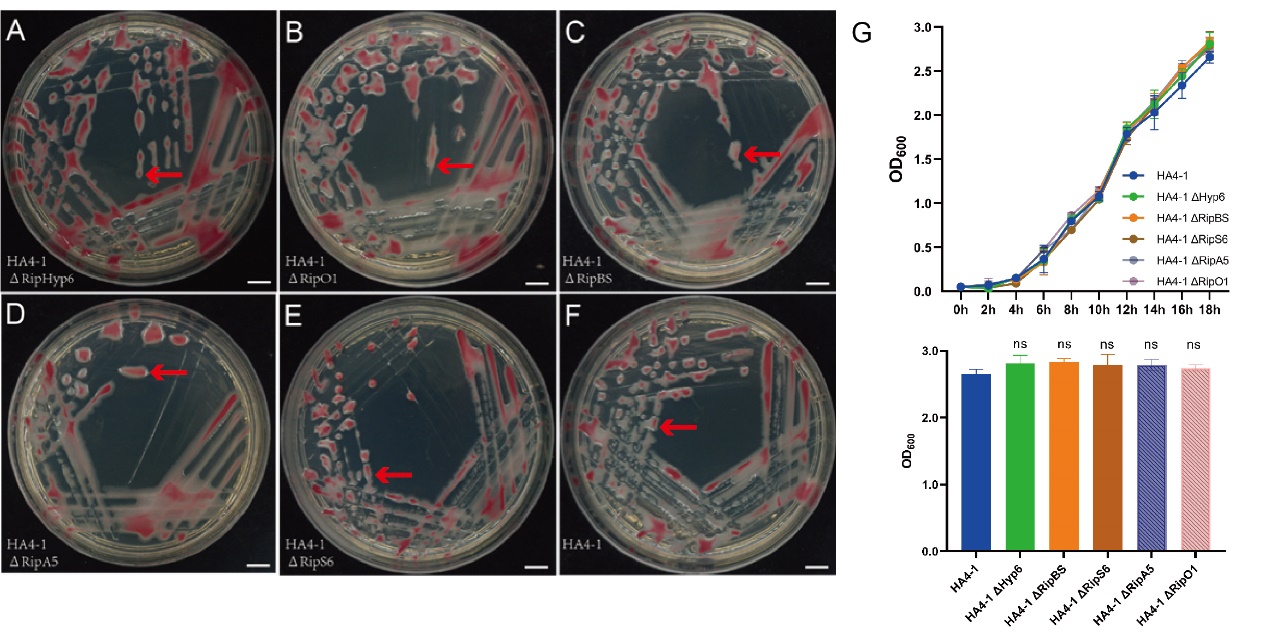


**Figure S4. HA4-1 mutant strains still triggered the HR of ALB28-1 leaves.** All strains were resuspended at OD_600_=0.1 (≈1 × 10^8^ CFU/mL) and infiltrated into 12 ALB28-1 leaves. The experiments were repeated at least three times with similar results. The white scale bar indicates 1 cm. The photo was taken at 3 dpi.


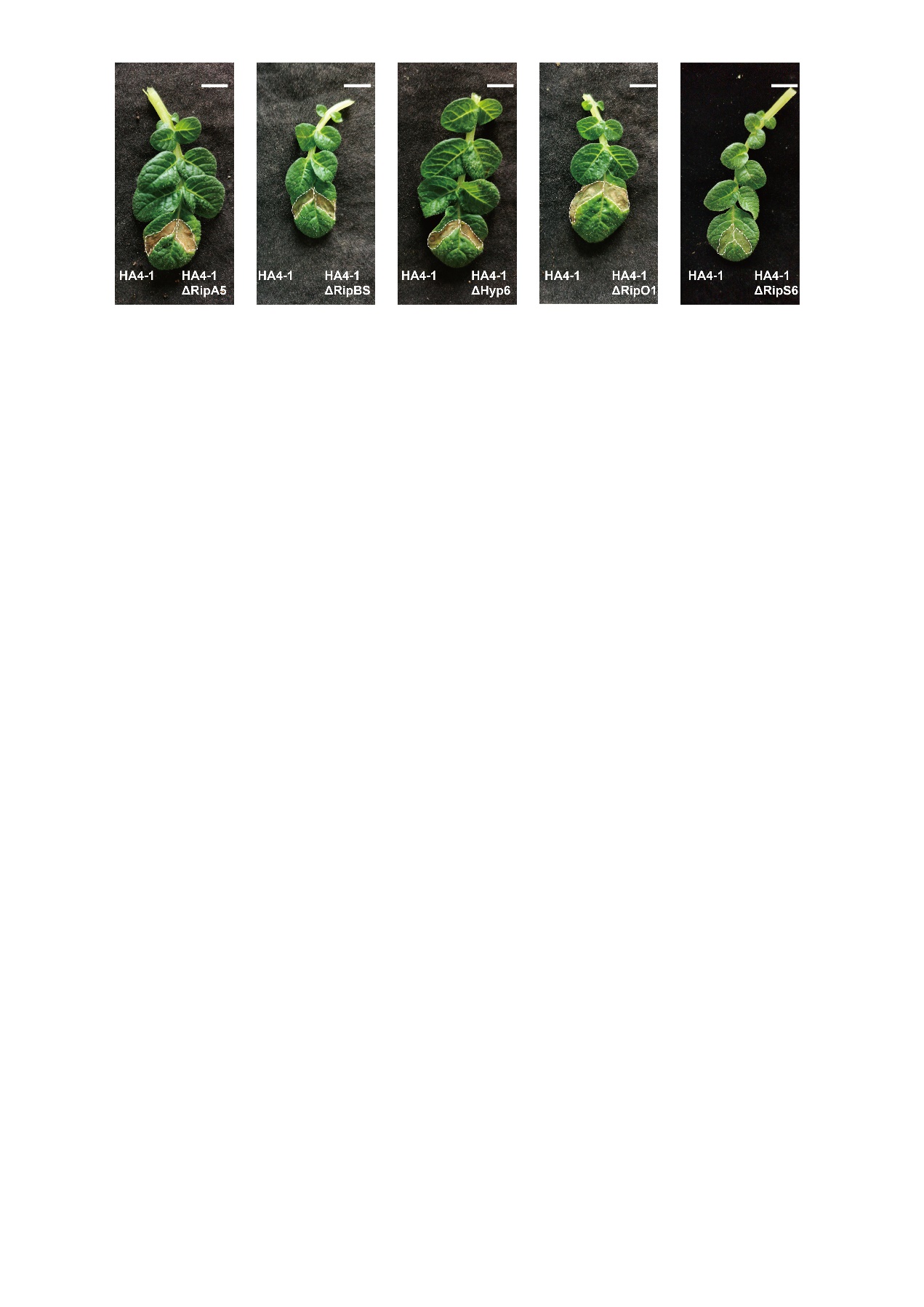


**Figure S5. The symptoms of susceptible host *Solanum tuberosum L.* cultivar E3 inoculated with *R. s* strains at 12 dpi. (A)** E3 showed wilting symptoms when inoculated with HA4-1 (WT) and HA4-1 mutants HA4-1 ΔRipS6, HA4-1 ΔRipBS. **(B)** E3 showed wilting symptoms when inoculated with HZAU091(WT), HZAU091::RipBS, and HZAU091::RipS6. The experiments were repeated three times with similar results. All strains were resuspended at OD_600_=0.1 (≈1 × 10^8^ CFU/mL).


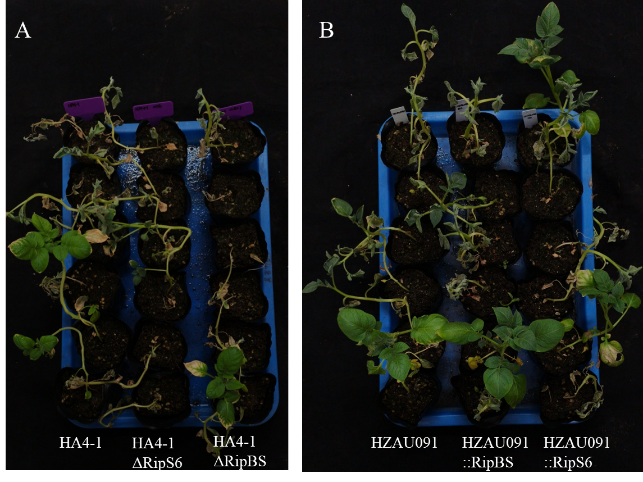


**Figure S6. Amino acid sequence alignment of RipBS, XopAC (GenBank: AFP74845) and RipAC (GenBank: NZ_CP021763.1).** The yellow boxes indicate the conserved LRR domain of RipBS. The orange boxes indicate the conserved Fic domain of RipBS.


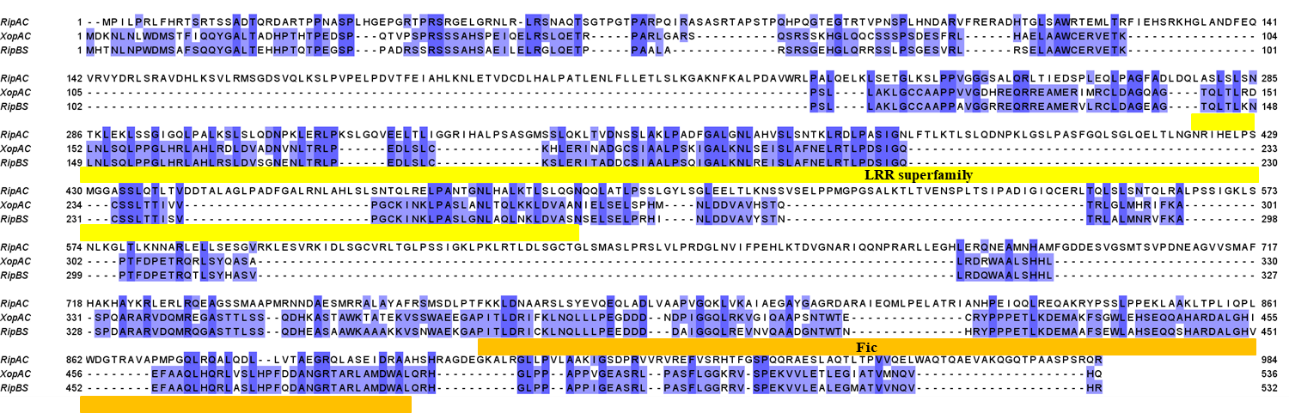

Supplement: Supplementary file 2 [file DataSheet_1.docx]
